# A Carga Aterosclerótica é o Caminho para Eventos Cardiovasculares

**DOI:** 10.36660/abc.20220554

**Published:** 2022-08-25

**Authors:** Tannas Jatene, Jordana Pires Mendonça, Vinicius Daher Vaz, Fabrício Ribeiro Las Casas, Rogério Lobo de Andrade Las Casas

**Affiliations:** 1 Hospital Israelita Albert Einstein Goiânia GO Brasil Hospital Israelita Albert Einstein, Goiânia, GO – Brasil; 2 Hospital do Coração Anis Rassi Goiânia GO Brasil Hospital do Coração Anis Rassi, Goiânia, GO – Brasil

**Keywords:** Aterosclerose, Doença da Arterial Coronariana, Fibrilação Atrial, Infarto do Miocárdio, Intervenção Coronária Percutânea

A doença cardiovascular aterosclerótica ainda é uma das principais causas de morbidade e mortalidade em todo o mundo e seus fatores de risco já foram identificados. Dislipidemia, hipertensão arterial, tabagismo, diabetes e adiposidade estão frequentemente presentes, principalmente em associação, em pacientes com doença arterial coronariana (DAC).^1 , 2^ Identificar pacientes com risco de desenvolver DAC e apresentar síndromes coronarianas agudas é de grande valia, e muitos escores foram criados com esse objetivo, como o escore de risco de Framingham, o escore ASSIGN e o escore de risco QRISK^R^2.

O escore CHA_2_DS_2_VASc, que compreende insuficiência cardíaca congestiva (C), hipertensão (H), idade 75 (A_2_), diabetes (D), acidente vascular cerebral ou ataque isquêmico transitório (S_2_), doença vascular (V), idade 65-74 anos (A) e sexo masculino (Sc), foi originalmente desenvolvido para prever o risco de acidente vascular cerebral em pacientes com fibrilação atrial. Recentemente, o escore CHA_2_DS_2_VASc também foi associado a eventos adversos maiores em pacientes com infarto do miocárdio com supradesnivelamento do segmento ST (IAMCSST),^3^ síndromes coronárias agudas sem supradesnivelamento do segmento ST^4 , 5^ e em pacientes com doença isquêmica crônica estável.^6^ Além disso, Tasolar et al.,^4^ e Chua et al.,^5^ demonstraram sua associação com a gravidade da doença arterial coronariana.

O escore de SYNTAX residual (rSS) foi projetado para quantificar a incompletude da revascularização após intervenção coronária percutânea (ICP), calculando o escore SYNTAX (Synergy Between PCI With Taxus and Cardiac Surgery) restante, uma medida angiográfica quantitativa de gravidade e complexidade anatômica após uma ICP. Um rSS alto foi associado a um prognóstico ruim em 30 dias e 1 ano.^7^

Neste número dos Arquivos Brasileiros de Cardiologia, Kalkan et al.,^8^ demonstraram pela primeira vez a associação entre o escore CHA_2_DS_2_VASc e rSS em 688 pacientes submetidos à ICP primária após IAMCSST.^8^ Embora pareça óbvio encontrar uma relação entre um escore que inclua alguns dos principais fatores de risco para aterosclerose (CHA_2_DS_2_VASc) e a própria carga aterosclerótica (rSS), chama a atenção para a discussão relevante e contemporânea sobre a importância da gravidade da doença aterosclerótica.Possivelmente, a carga aterosclerótica é o mecanismo por trás da associação do escore CHA_2_DS_2_VASc e eventos cardiovasculares encontrados em muitos estudos anteriores.^3 , 5 , 6^

Diretrizes internacionais sugerem a documentação de isquemia antes de procedimentos invasivos eletivos para tratamento de doença arterial coronariana, seja por eletrocardiograma de esforço, ecocardiograma de estresse, cintilografia de perfusão miocárdica ou ressonância magnética cardíaca. Além disso, testes funcionais invasivos, como reserva de fluxo fracionada, são recomendados antes da revascularização se a isquemia não invasiva não for demonstrada.^9^ Controversamente, a superioridade das avaliações anatômicas sobre os testes de isquemia tem sido repetidamente demonstrada em diferentes cenários. Em um estudo randomizado de 3.283 pacientes, Singh et al.,^10^ mostraram que a angiotomografia computadorizada (TC) teve uma associação mais forte com doença cardíaca coronária de 5 anos, morte ou infarto do miocárdio (IAM) não fatal do que o eletrocardiograma de esforço.^10^ Da mesma forma, Hoffmann et al.,^11^ demonstraram em um estudo randomizado de 9.102 pacientes que a angiotomografia teve uma capacidade discriminatória maior do que o teste funcional na previsão de eventos cardiovasculares.^11^ Em uma subanálise do estudo COURAGE, a carga anatômica avaliada pela angiografia coronariana foi um preditor consistente de morte, IAM e síndromes coronarianas agudas sem supradesnivelamento do segmento ST, enquanto a carga isquêmica não foi.^12^ Uma subanálise recente do estudo ISCHEMIA revelou que a gravidade da DAC, avaliada pela angiotomografia, foi um preditor altamente significativo de mortalidade por todas as causas, morte cardiovascular e IAM, tanto espontâneo quanto periprocedimento; novamente, a gravidade da isquemia não foi associada a eventos adversos.^13^

A lógica por trás dos testes anatômicos serem superiores aos testes de isquemia é simples. O IAM espontâneo ocorre quando uma placa obstrutiva ou não obstrutiva se rompe; os testes funcionais identificam apenas placas obstrutivas, enquanto a angiografia, invasiva ou por TC, identifica placas grandes e pequenas, que podem erodir ou romper, causando IAM e possivelmente a morte. Consequentemente, se você tem mais placas, tem maior chance de instabilidade em uma delas e maior risco de eventos cardiovasculares. Um benefício teórico da cirurgia de revascularização miocárdica sobre a ICP baseia-se na ideia de que o enxerto protege longos segmentos de placas coronárias proximais, e o paciente estaria protegido de IAM se qualquer uma dessas se rompe, enquanto os stents protegem apenas o segmento com stent.^14^

Em conclusão, Kalkan et al.,^8^ demonstraram que pacientes com maior escore CHA_2_DS_2_VASc tem maior rSS, o que significa maior gravidade da DAC. Com base em evidências recentes, esses pacientes podem, consequentemente, estar em maior risco de ruptura da placa e eventos cardiovasculares, como infarto do miocárdio ou morte e, portanto, se beneficiariam de terapias estabilizadoras de placa mais agressivas. Ao prever a carga aterosclerótica, o escore CHA_2_DS^2^ VASc pode ser mais uma ferramenta para predição de risco em pacientes com DAC.
