# Supplementary material for: A Carga Aterosclerótica é o Caminho para Eventos Cardiovasculares
Source: Arq Bras Cardiol. 2022 Aug 25;119(3):400–1. [Article in Portuguese] doi: 10.36660/abc.20220554 (PMC9438547; doi:10.36660/abc.20220554)
Supplement: Translation

# Translation: Atherosclerotic Burden is the Highway to Cardiovascular Events

**Authors:** Tannas Jatene, Jordana Pires Mendonça, Vinicius Daher Vaz, Fabrício Ribeiro Las Casas, Rogério Lobo de Andrade Las Casas

**Affiliations:** 1 Brazil Hospital Israelita Albert Einstein, Goiânia, GO – Brazil; 2 Brazil Hospital do Coração Anis Rassi, Goiânia, GO – Brazil

**Keywords:** Atherosclerosis, Coronary Artery Disease, Atrial Fibrillation, Myocardial Infarction, Percutaneous Coronary Intervention

Atherosclerotic cardiovascular disease is still a major cause of morbidity and mortality worldwide, and its risk factors have already been identified. Dyslipidemia, high blood pressure, cigarette smoking, diabetes and adiposity are frequently present, mostly in combination, in patients with coronary artery disease (CAD).^1 , 2^ Identifying patients at risk for developing CAD and presenting acute coronary syndromes is of great value, and many scores were created with this aim, such as the Framingham risk score, the ASSIGN score and the QRISK^R^2 risk score.

CHA_2_DS_2_VASc score, which comprises congestive heart failure (C), hypertension (H), age ≥ 75 (A_2_), diabetes (D), stroke or transient ischemic attack (S_2_), vascular disease (V), age 65-74 years (A) and male gender (Sc), was originally developed to predict stroke risk in patients with atrial fibrillation. Recently, the CHA_2_DS_2_VASc score was also associated with major adverse events in patients with ST-segment elevation myocardial infarction (STEMI)^3^ non-ST elevation acute coronary syndromes^4 , 5^ and in patients with chronic stable ischemic disease.^6^ Moreover, Tasolar et al.^4^ and Chua et al.^5^ demonstrated its association with coronary artery disease severity.

Residual syntax score (rSS) was designed to quantify the incompleteness of revascularization after percutaneous coronary intervention (PCI), calculating the remaining SYNTAX (Synergy Between PCI With Taxus and Cardiac Surgery) score, a quantitative angiographic measure of anatomic severity and complexity after a PCI. A high rSS was associated with a poor 30-day and 1-year prognosis.^7^

In this issue of *Arquivos Brasileiros de Cardiologia* , Kalkan et al.^8^ demonstrated for the first time the association between CHA_2_DS_2_VASc score and rSS in 688 patients who underwent primary PCI after STEMI.^8^ Although it seems obvious to find a relationship between a score that includes some of the main risk factors for atherosclerosis (CHA_2_DS_2_VASc) and the atherosclerotic burden itself (rSS), it brings our attention to the relevant and contemporary discussion about the importance of the severity of atherosclerosis. Possibly, the atherosclerotic burden is the mechanism behind the association of CHA_2_DS_2_VASc score and cardiovascular events found in many previous studies^3 , 5 , 6^

International guidelines suggest the documentation of ischemia before elective invasive procedures to treat coronary artery disease, either by exercise electrocardiogram testing, stress echocardiography, single-photon emission computed tomography or cardiac magnetic resonance. Moreover, invasive functional testing, such as fractional flow reserve, is recommended before revascularization if non-invasive ischemia is not demonstrated.^9^ Controversially, the superiority of anatomic assessments over ischemia testing has been repeatedly demonstrated in different scenarios. In a randomized trial of 3283 patients, Singh et al.^10^ showed that computed tomography (CT) angiography had a stronger association with 5-year coronary heart disease, death, or non-fatal myocardial infarction (MI) than exercise electrocardiogram.^10^ Similarly, Hoffmann et al.^11^ demonstrated in a randomized trial of 9102 patients that CT angiography had a greater discriminatory ability than functional testing in predicting cardiovascular events^11^ . In a sub-analysis of the COURAGE trial, anatomic burden assessed by coronary angiography was a consistent predictor of death, MI and non-ST segment elevation acute coronary syndromes, whereas ischemic burden was not.^12^ A recent sub-analysis of the ISCHEMIA trial revealed that CAD severity, evaluated by CT angiography, was a highly significant predictor of all-cause mortality, cardiovascular death and MI, both spontaneous and periprocedural; again, ischemia severity was not associated with adverse events.^13^

The rationale behind anatomic tests being superior to ischemia testing is simple. Spontaneous MI occurs when either an obstructive or non-obstructive plaque ruptures; functional tests identify only obstructive plaques, whereas angiography, invasive or CT, identifies big and small plaques, which can erode or rupture, causing MI and possibly death. Consequently, if you have more plaques, you have a bigger chance of instability in one of those and a higher risk of cardiovascular events. One theoretical benefit of coronary artery bypass graft over PCI relies on the idea that the graft bypasses long segments of proximal coronary plaques, and the patient would be protected from MI if any of those ruptures, whereas the stents protect only the stented segment.^14^

In conclusion, Kalkan et al.^8^ demonstrated that patients with higher CHA_2_DS_2_VASc score have higher rSS, which means higher CAD severity. Based on recent evidence, those patients might consequently be at higher risk of plaque rupture and cardiovascular events, such as MI or death, and therefore would benefit from more aggressive plaque stabilizing therapies. By predicting atherosclerotic burden, the CHA_2_DS_2_VASc score might be another tool for risk prediction in patients with CAD.
